# *In Vitro* Comparison of Dynesys, PEEK, and Titanium Constructs in the Lumbar Spine

**DOI:** 10.1155/2015/895931

**Published:** 2015-08-17

**Authors:** Matthew S. Yeager, Daniel J. Cook, Boyle C. Cheng

**Affiliations:** ^1^Department of Neurosurgery, Allegheny Health Network, Pittsburgh, PA 15212, USA; ^2^Department of Neurosurgery, Drexel University College of Medicine, Pittsburgh, PA 15212, USA

## Abstract

*Introduction*. Pedicle based posterior dynamic stabilization systems aim to stabilize the pathologic spine while also allowing sufficient motion to mitigate adjacent level effects. Two flexible constructs that have been proposed to act in such a manner, the Dynesys Dynamic Stabilization System and PEEK rod, have yet to be directly compared *in vitro* to a rigid Titanium rod.* Methods*. Human lumbar specimens were tested in flexion extension, lateral bending, and axial torsion to evaluate the following conditions at L4-L5: Intact, Dynesys, PEEK rod, Titanium rod, and Destabilized. Intervertebral range of motion, interpedicular travel, and interpedicular displacement metrics were evaluated from 3rd-cycle data using an optoelectric tracking system.* Results*. Statistically significant decreases in ROM compared to Intact and Destabilized conditions were detected for the instrumented conditions during flexion extension and lateral bending. AT ROM was significantly less than Destabilized but not the Intact condition. Similar trends were found for interpedicular displacement in all modes of loading; however, interpedicular travel trends were less consistent. More importantly, no metrics under any mode of loading revealed significant differences between Dynesys, PEEK, and Titanium.* Conclusion*. The results of this study support previous findings that Dynesys and PEEK constructs behave similarly to a Titanium rod *in vitro*.

## 1. Introduction

Lumbar fusion with rigid posterior instrumentation remains the standard treatment for various lumbar degenerative pathologies. However, fixation of spinal segments with rigid constructs such as Titanium rods and screws is thought a potential culprit in the development of adjacent segment degeneration [1–4]. Pedicle based posterior dynamic stabilization (PDS) has been proposed as a motion preserving alternative to fusion, primarily based on the perceived potential to internally brace the pathologic spinal segment while also restoring near-normal kinematic behavior, thus mitigating iatrogenic degeneration and adjacent level effects. Limited data exists, however, supporting their efficacy as standalone devices.

**Figure 1:**
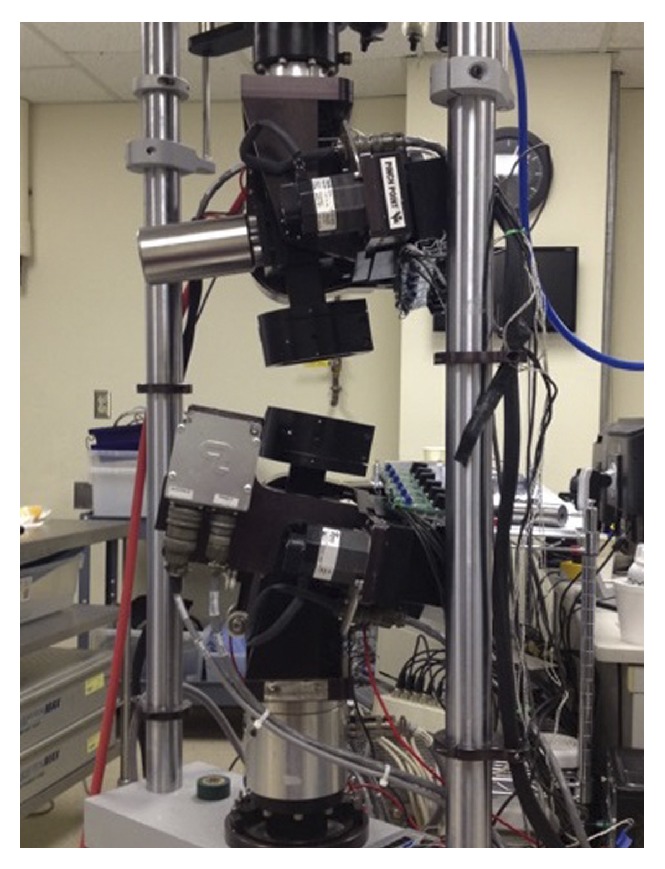
Biomechanical test frame. Lateral view of the test frame used with no specimen loaded. The cups used to secure specimens to the frame can be seen slightly angled, which would result in flexion if the specimen was loaded with the anterior column facing the left.

The Graf ligament, introduced in 1992, utilizes braided polyester cords looped around pedicle screws to stabilize the spine [5]. Clinical studies are inconclusive as to its effectiveness, potentially because it provides stability primarily in flexion [6, 7]. A more recently developed flexible device, the Dynesys Dynamic Stabilization System (Zimmer Spine, Warsaw, IN), consists of both a polycarbonate urethane spacer and a tensioned polyethylene terephthalate cord (Figure 1).* In vitro* biomechanical studies on the Dynesys (DYN) system have shown that it acts similarly to a rigid rod by substantially limiting motion in the sagittal and coronal planes yet does little to restrict axial rotation [8–13]. Motion at levels adjacent to DYN when compared to a rigid rod has also been evaluated* in vitro,* and no significant differences have been detected [8, 14]. Further, DYN hybrid constructs (Titanium rod with DYN at the superior or inferior adjacent level) have been shown to demonstrate little difference in cadaveric stabilization compared to two-level rigid fixation [14, 15]. Many clinical studies on the Dynesys system have been conducted; however they mostly indicate noninferior or equivocal outcomes compared to traditional fusion or nonfusion techniques [16–24]. One such investigation reported DYN instrumentation after nucleotomy reduced index level disc degeneration at a mean follow-up of 34 months compared to nucleotomy alone [25]. While both groups showed a significant improvement in pain and activities of daily living at 3 months, only the nucleotomy group showed an additional significant improvement at subsequent follow-up.

Polyetheretherketone (PEEK) rods have been proposed as an alternative to rigid fusion primarily due to having an elastic modulus that is much lower than that of Titanium [27]. However, although benchtop evaluations show increased flexibility of PEEK rods compared to Titanium, various cadaveric biomechanical investigations of PEEK rods have yet to demonstrate stabilization characteristics that are significantly different compared to similar rods constructed of Titanium [27–31]. Clinical investigations are particularly sparse. Highsmith et al. reported on 1 case utilizing PEEK as a standalone fixator; however outcomes were not reported other than the initial reduction of spondylolisthesis [31].

To the authors' knowledge, no studies to date have directly compared the* in vitro* biomechanical behavior of PEEK, DYN, and Titanium (TI) rods. While traditional parameters such as range of motion (ROM) and finite helical axis (FHA) provide valuable information about the motion of the affected functional spinal unit (FSU), additional metrics have been defined that may elucidate kinematic differences in the spine relative to the pedicle screw head. These metrics, interpedicular travel (IPT) and interpedicular displacement (ID), have been shown to provide information more sensitive to device motion and can expose subtle differences between constructs that had previously been unattainable [32]. This study was designed to evaluate whether standalone DYN and/or PEEK constructs significantly restrict motion compared to the Intact condition and further elucidate the* in vitro* biomechanical characteristics of these constructs when compared to TI in the lumbar spine.

## 2. Materials and Methods

### 2.1. Specimen Preparation

Seven fresh-frozen human lumbar T12–S1 segments (mean age ± standard deviation (SD) = 54 ± 8 years) were stripped of all soft tissues with the exception of the osteoligamentous structures. The anterior longitudinal ligament was also removed from each vertebral body to facilitate fixation of tracking hardware. Each specimen was scanned prior to testing with computerized tomography (CT) and after testing with Dual-energy X-ray absorptiometry (DEXA) to acquire bone mineral density (BMD) of the untreated levels. CT and DEXA results revealed no gross pathology or substantial loss in BMD (mean ± SD = 0.92 ± 0.15 g/cm^2^).

The cranial and caudal ends of each specimen were potted in aluminum rings using a thermosetting potting resin (Bondo, 3M, Atlanta, GA). Wood screws were inserted into each vertebra to ensure adequate fixation within the potting resin. A rigid aluminum tracking body containing four active light emitting diodes was then affixed to the anterior surface of each vertebra with potting resin and aluminum sheet metal screws. The potting resin was allowed to cure for a minimum of 24 hours prior to any testing. All specimens were double bagged and stored at −20°C when not in use. Prior to testing, specimens were allowed to thaw at 5°C and brought to room temperature. Saline soaked gauze was wrapped around each specimen to prevent dehydration.

### 2.2. Testing and Instrumentation

Testing consisted of a standard flexibility protocol, carried out on a biaxial test frame (Figure 1) mounted with counteracting superior and inferior flexion-extension (FE) and lateral bending (LB) motors (Bose, Smart Test Series, Eden Prairie, MN). Pure moment sinusoidal loading was employed at 0.005 Hz to load limits of ±7.5 Nm in FE, LB, and axial torsion (AT), as well as axial compression (AC) to 150 N. The applied loads were cycled 3 times for each mode and third-cycle data was used for analysis. No axial preload was applied in this study.

**Figure 2:**
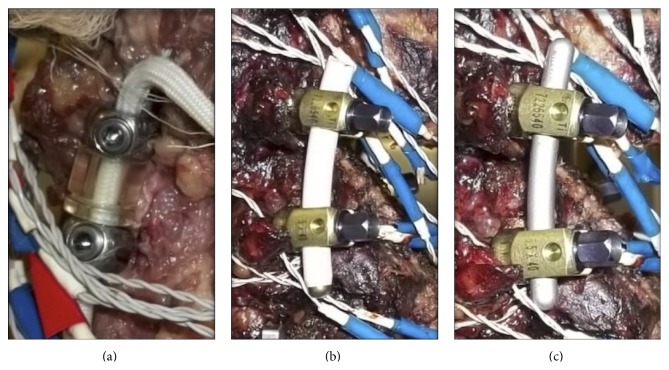
Dynesys, PEEK, and Titanium constructs. Instrumented at left L4-L5 index level. From left to right: posterior view of Dynesys construct; lateral view of PEEK construct; lateral view of Titanium construct.

All specimens were subjected to the following treatments at the L4-L5 index level: Intact, Destabilized (laminectomy and partial foraminotomy), DYN (12.0 mm spacer, 5.5 mm diameter screw, Zimmer Spine, Warsaw, IN), PEEK (6.0 × 6.9 mm oblong rod, 6.5 mm diameter screw, Medtronic, Memphis, TN), and TI (5.5 mm diameter rod, 6.5 mm diameter screw, CD-Horizon, Medtronic, Memphis, TN). The Destabilization procedure was conducted prior to the implantation of the DYN construct; however testing of the Destabilized condition was conducted last in order to prevent loss of data caused by potential damage to the specimen. Further, the PEEK and TI treatments were carried out in randomized order to mitigate influences due to specimen degradation or loss of screw purchase. Randomization with regard to the DYN construct was not carried out because the smaller screw diameter (5.5 mm) would have resulted in inadequate purchase if implanted after the PEEK or Titanium constructs, both of which utilized a screw with larger diameter (6.5 mm). Screw implantation was carried out by first decorticating the pedicle lateral to the facet joint, with care to not disrupt the facet capsule. A curved pedicle probe was then used to create a path for the DYN screw, after which a ball probe was utilized to confirm the integrity of the surrounding bone. After implantation and testing, the DYN screw was removed and the larger diameter PEEK/TI screw was then inserted along the same path. Due to the larger diameter of the screw, it is unlikely that the screw implantation method and sequence had any effect on the results. Pretensioning of the DYN cord was carried out to 300 N, in accordance with the surgical technique. Each system can be seen in Figure 2.

**Figure 3:**
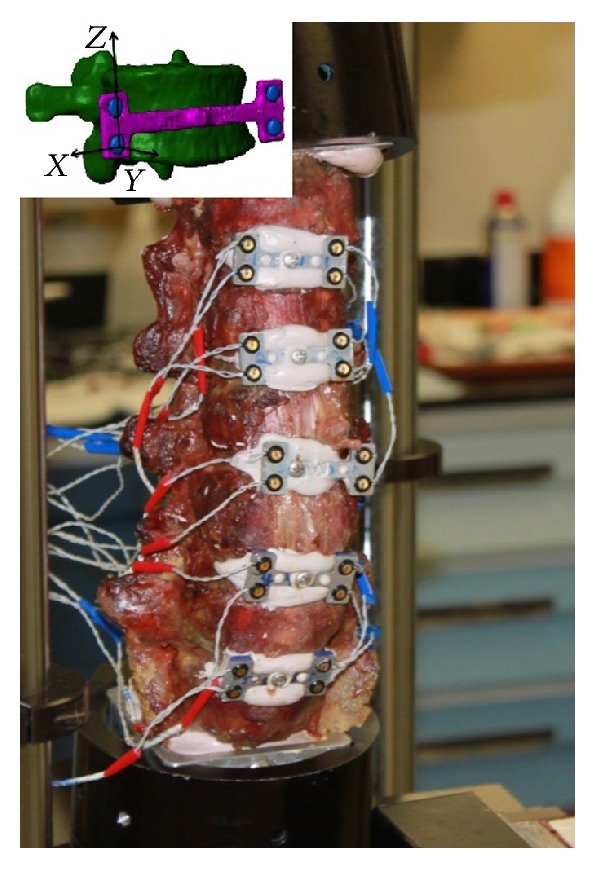
Spine with tracking bodies. Tracking bodies are affixed to the anterior column for 3D tracking of vertebral bodies throughout testing. The upper left of the figure shows a virtual representation of the local coordinate frame defined on a tracking body.

Kinematic parameters were tracked using an Optotrak Certus motion capture system with a manufacturer stated accuracy of 0.1 mm (Optotrak, Northern Digital Instruments, Waterloo, ON, Canada). Range of motion (ROM) and finite helical axis (FHA) measurements for each intervertebral level were obtained from the anteriorly placed vertebral tracking bodies (Figure 3). ROM was calculated as the range of the Euler angle corresponding to flexion-extension, lateral bending, and axial rotation modes of loading based on vertebral tracking body motion. During axial compression, ROM was defined as the range of translation in the axial direction of each FSU. FHA was also calculated based on the vertebral tracking body motion, based on the method of Reuleaux Woltring et al. [33].

**Figure 4:**
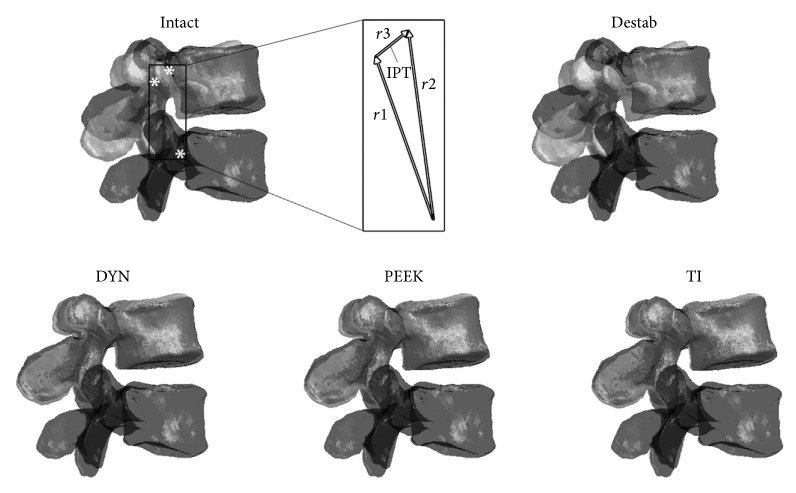
IPT and segmented vertebrae. Representative L4-L5 FSU showing displacement of superior vertebra at maximum flexion and extension angles for each treatment. (IPT = interpedicular travel; Destab = Destabilized; DYN = Dynesys; TI = Titanium rod.)

Additionally, the head of each pedicle screw was digitized relative to its respective vertebral tracking body and virtually tracked throughout testing (e.g., the heads of the left and right L4 pedicle screws were independently digitized relative to the anterior L4 tracking body). This allowed for the acquisition of IPT and ID measurements. IPT represents the magnitude of the vector describing displacement of adjacent pedicles at the index level between peak loading conditions (Figure 4). This quantifies the magnitude of linear travel of the superior pedicle landmark relative to inferior pedicle. ID on the other hand represents the change in magnitude of interpedicular distance throughout a given ROM and provides information more specific to axial distraction of a device. This describes the change in distance between pedicle landmarks and does not necessarily describe the magnitude of relative translation between them in all situations as does IPT. These metrics have the potential to provide a more complete characterization of PDS constructs and have been previously described in greater detail [32].

A mixed-effects' analysis of variance with Bonferroni-corrected post hoc analysis was conducted to elucidate significant differences between treatment conditions. All of the statistical analysis was performed using SPSS 18 (SPSS Inc., Chicago, IL).

## 3. Results

No statistically significant differences were observed between any of the instrumented conditions in any mode of loading. Significant differences compared to Intact and Destabilized conditions are described below, while mean and standard deviation values for all metrics can be found in Table 1.

### 3.1. ROM

Statistically significant differences in ROM compared to Intact and Destabilized conditions were detected for the instrumented conditions during FE, LB, and AT (Figure 3). In FE, normalized to the Intact condition, DYN, PEEK, and TI all showed significant reductions of 57%, 61%, and 65% (*p* ≤ 0.001). In LB, 48%, 53%, and 65% (*p* ≤ 0.001) reductions were observed. In AT, all 3 treatments were significantly reduced compared to the Destabilized condition (*p* ≤ 0.014); however none showed a significant reduction compared to the Intact condition. AT ROM increased 5% for DYN and was reduced by 20% and 27% for PEEK and TI, respectively. AC results showed little difference between treatments, and no significant differences were found between DYN, PEEK, and TI.

### 3.2. IPT

Compared to the Intact condition, a statistically significant reduction of 37% (*p* ≤ 0.001) was found in FE for the DYN condition, as well as a 19% increase for the Destabilized condition (*p* ≤ 0.028) (Figure 4). No other significant differences in IPT compared to Intact condition were found across treatments. All instrumented conditions showed a significant reduction compared to the Destabilized condition in FE (*p* ≤ 0.028) and AC (*p* ≤ 0.047); however only DYN was significantly reduced in LB (*p* ≤ 0.012). Further, no significant reductions were found across any treatments in AT, and no significant reductions were found between DYN, PEEK, and TI.

### 3.3. ID

**Figure 5:**
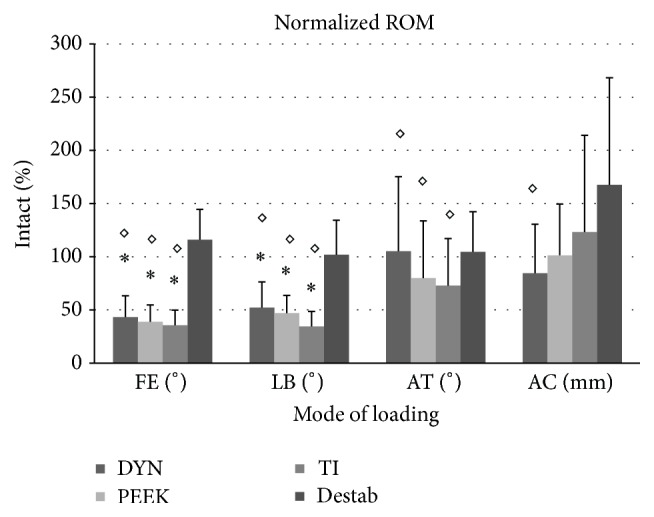
Normalized range of motion at L4-L5. (DYN = Dynesys; TI = Titanium; FE = flexion-extension; LB = lateral bending; AT = axial torsion; AC = axial compression; *∗* = significant decrease compared to the Intact condition; ◊ = significant decrease compared to Destabilized condition; † = significant increase compared to the Intact condition.)

**Figure 6 fig6:**
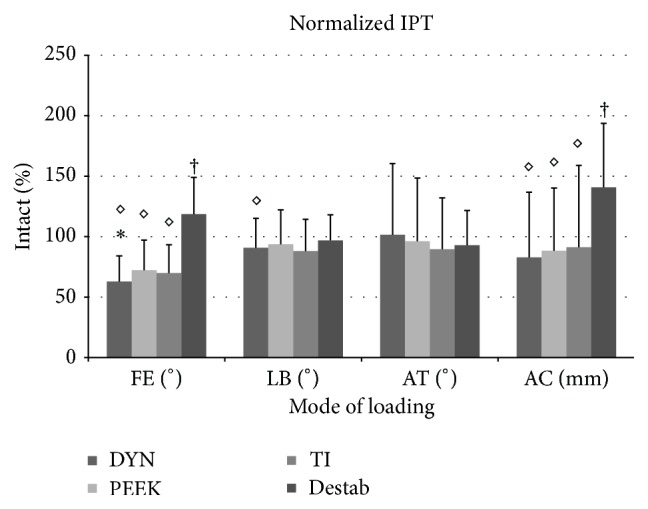
Normalized left IPT at L4-L5. (IPT = interpedicular travel; DYN = Dynesys; TI = Titanium; FE = flexion-extension; LB = lateral bending; AT = axial torsion; AC = axial compression; *∗* = significant decrease compared to the Intact condition; ◊ = significant decrease compared to the Destabilized condition; † = significant increase compared to the Intact condition.)

**Figure 7 fig7:**
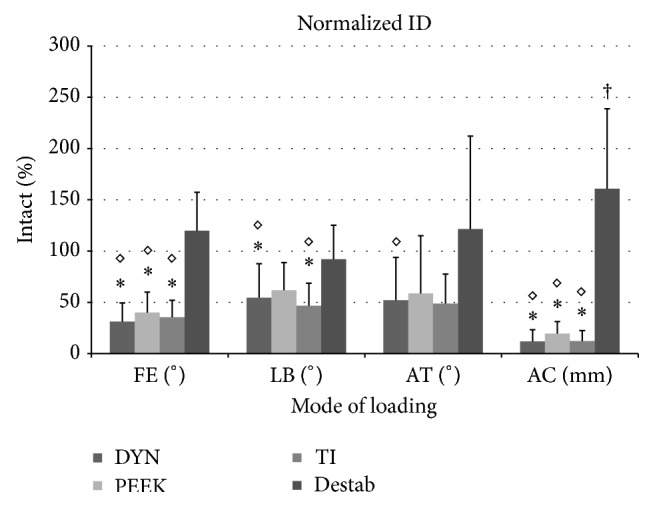
Normalized left ID at L4-L5. (ID = interpedicular displacement; DYN = Dynesys; TI = Titanium; FE = flexion-extension; LB = lateral bending; AT = axial torsion; AC = axial compression; *∗* = significant decrease compared to the Intact condition; ◊ = significant decrease compared to the Destabilized condition; † = significant increase compared to the Intact condition.)

In FE ID no statistically significant differences were detected between instrumented conditions or between Intact and the Destabilized conditions (Figure 5) [Fig fig7]. DYN, PEEK, and TI conditions showed a significant reduction (69%, 60%, and 65% of Intact, resp.) with respect to both Intact and Destabilized conditions (*p* ≤ 0.001). In LB no statistically significant differences were detected between instrumented conditions or between Intact and Destabilized conditions. DYN and TI showed significant reductions of 45% and 53% compared to the Intact condition (*p* ≤ 0.007 and 0.016, resp.), while DYN and TI showed a significant reduction compared to the Destabilized condition (*p* ≤ 0.001 and 0.012, resp.). Only DYN showed a significant reduction compared to the Destabilized condition in AT (*p* ≤ 0.041). In AC, the Destabilized condition showed a significantly greater ID compared to all other test conditions (*p* ≤ 0.042). DYN, PEEK, and TI exhibited reductions of 88%, 80%, and 88% ID compared to the Intact condition (*p* ≤ 0.001). Again, no significant reductions were found between DYN, PEEK, and TI.

### 3.4. Finite Helical Axis

Each instrumented treatment resulted in a posterior shift of the FHA with respect to both the Intact and Destabilized condition in FE (*p* < 0.005), and a significant inferior shift in the FHA was observed for DYN with respect to the Intact and Destabilized conditions (*p* = 0.008 and 0.011, resp.). In LB a significant inferior shift in the FHA was detected in both the PEEK and TI conditions with respect to the Intact condition (*p* = 0.022 and 0.047, resp.). No further statistically significant FHA differences were detected between any of the treatment groups in FE, LB, or AT.

## 4. Discussion

The current study compared the* in vitro* biomechanical performance of DYN, PEEK, and TI constructs in the human cadaveric lumbar spine. The ROM and FHA results echo those of previous studies: DYN and PEEK constructs, when used in a standalone application, perform similarly to TI rods. Further, no significant differences in metrics more specific to device motion, IPT and ID, were observed between constructs. The data presented further demonstrates that devices semicompliant in bending will not necessarily provide significantly greater motion in the affected FSU.

A particularly relevant parameter in this study is ID, which is a direct measurement of axial displacement and should correlate to axial stiffness of the device inserted between the pedicles. It is well known that rigid pedicle fixation results in a posterior shift in the center of rotation during FE [9, 34]. In order to restore sagittal plane motion characteristic of a healthy FSU, the design of a PDS device should focus on allowing sufficient ID in order to maintain a center of rotation comparable to the healthy FSU. In efforts to obtain the necessary stiffness required for dynamic implants to allow sufficient motion* in vivo*, finite element models (FEMs) have been employed. Rohlmann et al. used a validated FEM to compare a dynamic implant to rigid fixation and subsequently determine the effects of implant stiffness on ROM [35]. The dynamic implant was modeled with an axial stiffness of 200 N/mm, while that of the rigid fixator was significantly higher (83,000 N/mm). The effect of the rigid fixation was only slightly more pronounced. Moreover, the study varied implant stiffness in discrete steps between 1 and 83,000 N/mm. This demonstrated that only very low axial stiffness values, less than 200 N/mm, may have a marked effect on FE ROM. Other studies have confirmed these results [26, 36]. The influence of axial stiffness on sagittal plane motion is further bolstered by data demonstrating that 80–90% of FE bending loads in the spine are transferred by a force pair between the construct and the anterior column, and only a small percentage is directly borne by implant bending [37].

A more comprehensive evaluation of implant stiffness by Schmidt et al. not only considered axial stiffness in the sagittal plane, but paired it with bending stiffness in FE, LB, and AT [26]. Axial stiffness was found to be primarily responsible for reducing ROM in FE; however bending stiffness contributed more substantially in LB and AT. Further,* in vitro* pure moment testing to load limits of 7.5 Nm in each mode was used to compare reductions in ROM. For this, the DSS construct (Paradigm Spine, Wurmlingen, Germany), reportedly designed to match the stiffness of the FEM, and a rigid Titanium rod were used. Agreement was observed between cadaveric and FEMs in all modes but AT. More recently Schilling et al. compared the* in vitro* stabilization effects of multiple PDS constructs, including both the DSS and DYN [13]. In a brief comparison of results from the latter and current studies, it can be seen that the DSS and DYN stabilize the spine very differently in AT compared to traditional rod-based constructs (Table 2). It is thought that this can be primarily attributed to device design; the ability of a device to resist shear force is a critical component in AT [13]. IPT, which in this mode of loading approximates the magnitude of translation between pedicles in the transverse plane, should correlate to shear force resistance. Again, despite the variation in device designs, no statistically significant differences were found in the current study (DYN, PEEK, and TI) for any mode of loading. Further work should be done to reconcile differences of the effect of axial, bending, and shear stiffness on ROM.

### 4.1. Limitations and Future Work

Implantation of the DYN construct required cutting the PCU spacers to a variable length and inserting them bilaterally between differently spaced pedicle screws. The spacer length is directly proportional to the native distance between pedicles as well as the location and angle of the pedicle screw insertion. The spacer length thus varied bilaterally in each specimen, as well as between specimens. It has been previously demonstrated by Niosi et al. that spacer length directly influences lordosis, FSU motion, and loading [9]. However, they evaluated different spacer lengths in the same specimen and thus distracted and tensioned the FSU beyond its neutral position. In the current study, all spacers were cut to fit and cords were pretensioned without affecting the position of the instrumented vertebra, as would have been done in the operative setting. Further, the PEEK and TI rods were also sized to fit; thus the variation in length existed in all treatment conditions.

The PCU spacers are also known to have lower stiffness at higher temperatures. Studies have attempted to attenuate this discrepancy by using custom spacers that are less stiff, or by using heating elements to warm the implant to the desired temperature. To the authors' knowledge, however, no studies to date have demonstrated a significant difference in outcomes between body temperature spacers compared to rigid fixation. The original PCU spacers used in this study were kept at room temperature, which corresponds to a stiffness value of approximately 243 N/mm, while the stiffness at body temperature is 136 N/mm as reported by Davis [38]. This body temperature stiffness is lower than the estimated threshold of 200 N/mm reported by previous studies. However, during FE the spacer is likely to only have an effect on extension because the PTE cord is the limiting factor in flexion. The effects in LB and AT are more difficult to predict given the opposing direction of forces within the left and right constructs during these modes of loading.

Interpedicular measurements in this study were obtained using digitized points on the pedicle screws which were tracked relative to the tracking hardware affixed to the anterior surface of the vertebral body. Greater accuracy could have been achieved if these points had been digitized relative to a tracking body affixed directly to the pedicle screw. The vertebral body is not uniformly rigid and measurements obtained posteriorly may be slightly different than those on the anterior surface. Further, direct pedicle screw tracking can account for motion at the bone-screw interface that cannot otherwise be ascertained.

## 5. Conclusion

The results of this study confirm that DYN and PEEK constructs behave similarly to a TI rod* in vitro*. Additional metrics specific to device motion did not reveal any significant differences between constructs. Conversely, all constructs were different relative to Intact and Destabilized conditions. Future design should not only take into account maintenance of FSU motion about the rotational axes, but also allow adequate axial displacement between the pedicles, thus reducing posterior shift of the center of rotation caused by implantation. While current constructs may appearto be desirable as standalone dynamic stabilization systems over traditional rigid fusion, their ability to restore normal kinematic performance of the FSU, and subsequently prevent adjacent level degeneration, has yet to be established.

## Figures and Tables

**Table 1:** L4-L5 kinematic results (mean ± SD).

		Intact	DYN	PEEK	TI	Destabilized
ROM (°)	FE	12.47 ± 3.77	5.39 ± 2.50	4.83 ± 1.99	4.43 ± 1.78	14.45 ± 3.57
	LB	9.68 ± 2.40	5.05 ± 2.34	4.55 ± 1.60	3.33 ± 1.37	9.86 ± 3.14
	AT	4.45 ± 3.16	4.68 ± 3.12	3.55 ± 2.40	3.24 ± 1.97	4.65 ± 1.68
	AC	0.28 ± 0.16	0.23 ± 0.13	0.28 ± 0.13	0.34 ± 0.25	0.46 ± 0.28

IPT (mm)						
Left	FE	7.61 ± 1.49	4.78 ± 1.61	5.50 ± 1.89	5.32 ± 1.78	9.02 ± 2.31
	LB	9.51 ± 2.63	8.64 ± 2.32	8.92 ± 2.71	8.38 ± 2.50	9.22 ± 2.00
	AT	5.18 ± 2.99	5.26 ± 3.05	4.98 ± 2.71	4.64 ± 2.21	4.81 ± 1.49
	AC	1.04 ± 0.51	0.86 ± 0.56	0.92 ± 0.54	0.95 ± 0.70	1.47 ± 0.55
Right	FE	8.55 ± 2.47	5.85 ± 2.46	6.22 ± 2.41	6.06 ± 2.34	9.78 ± 2.86
	LB	8.85 ± 1.76	8.36 ± 1.96	7.99 ± 1.89	7.69 ± 1.80	9.38 ± 1.47
	AT	3.68 ± 1.65	4.09 ± 1.87	3.57 ± 1.62	3.44 ± 1.53	3.87 ± 1.19
	AC	1.11 ± 0.39	0.88 ± 0.55	0.94 ± 0.49	1.00 ± 0.65	1.69 ± 0.77

ID (mm)						
Left	FE	6.87 ± 1.90	2.15 ± 1.25	2.74 ± 1.38	2.44 ± 1.15	8.22 ± 2.59
	LB	5.97 ± 2.06	3.26 ± 1.97	3.69 ± 1.61	2.79 ± 1.32	5.51 ± 1.97
	AT	0.80 ± 0.64	0.42 ± 0.34	0.47 ± 0.45	0.39 ± 0.23	0.98 ± 0.73
	AC	0.79 ± 0.36	0.10 ± 0.09	0.16 ± 0.09	0.10 ± 0.08	1.28 ± 0.62
Right	FE	7.47 ± 2.29	2.26 ± 1.97	2.40 ± 0.84	2.17 ± 0.84	9.04 ± 2.96
	LB	5.26 ± 1.32	2.65 ± 1.75	2.36 ± 0.83	1.52 ± 0.57	5.87 ± 2.06
	AT	0.68 ± 0.49	0.55 ± 0.45	0.68 ± 0.84	0.60 ± 0.67	1.08 ± 0.60
	AC	0.90 ± 0.35	0.13 ± 0.10	0.17 ± 0.11	0.10 ± 0.04	1.52 ± 0.85

ROM = range of motion; IPT = interpedicular travel; ID = interpedicular displacement; FE = flexion-extension; LB = lateral bending; AT = axial torsion; AC = axial compression; DYN = Dynesys; TI = Titanium rod.

**Table 2:** Reduction in ROM at L4-L5 compared to previous studies.

Reduction in ROM normalized to intact				
	Flex.	Ext.	LB	AT
DSS-FEM^*∗*^	51%	40%	30%	29%
DSS^*∗*^	54%	39%	45%	70%
DSS^†^	55%		46%	41%
DYN	57%		48%	−5%
DYN^†^	66%		60%	5%
PEEK	61%		53%	20%
TI	64%		66%	27%
RR^*∗*^	74%	62%	71%	26%
RR^†^	71%		76%	58%
RR-FEM^*∗*^	76%	66%	62%	48%

^*∗*^Indicating treatment from Schmidt et al. [26]; ^†^indicating treatment from Schilling et al. [13]; DSS-FEM = predicted dynamic stabilization from finite element model; DSS = Dynamic Stabilization System; DYN = Dynesys; TI = Titanium rod; RR = rigid rod; RR-FEM = predicted rigid stabilization from finite element model.
